# Cost‐effectiveness of uterine tamponade devices for the treatment of postpartum hemorrhage: A systematic review

**DOI:** 10.1002/ijgo.13393

**Published:** 2020-10-30

**Authors:** Joshua P. Vogel, Alyce N. Wilson, Nick Scott, Mariana Widmer, Fernando Althabe, Olufemi T. Oladapo

**Affiliations:** ^1^ Maternal, Child and Adolescent Health Program Burnet Institute Melbourne Vic. Australia; ^2^ School of Population Health and Preventative Medicine Monash University Melbourne Vic. Australia; ^3^ UNDP/UNFPA/UNICEF/WHO/World Bank Special Program of Research Development and Research Training in Human Reproduction (HRP) Department of Sexual and Reproductive Health and Research World Health Organization Geneva Switzerland

**Keywords:** Cost, Cost‐effectiveness, Postpartum hemorrhage, Uterine balloon tamponade, Uterine tamponade

## Abstract

**Background:**

Uterine tamponade is widely promoted for treating refractory postpartum hemorrhage (PPH); however, its cost‐effectiveness may vary depending on unit costs and setting.

**Objective:**

To review available data on cost‐effectiveness of uterine tamponade devices when used for PPH treatment.

**Search strategy:**

PubMed and EMBASE were searched (1980 to January 2020), as well as the National Health Services Economic Evaluation database from inception (1995) to March 2015.

**Selection criteria:**

Eligible studies were any type of economic evaluation, or effectiveness studies that provided cost or economic data.

**Data collection and analysis:**

Two reviewers independently screened studies, extracted data, and assessed quality.

**Main results:**

Eleven studies using a range of devices (condom catheter, uterine suction devices, Bakri, Inpress, Ellavi) were identified. Cost of condom catheter devices or kits ranged from US$0.64 to US$6, whereas purpose‐designed device costs were up to US$400. Two studies that took a health system perspective assessed the cost‐effectiveness of using uterine balloon tamponade and suggested that it was highly cost‐effective because of the low cost per disability‐adjusted life‐year averted, although both used effect estimates from case series.

**Conclusions:**

Evidence on the cost‐effectiveness of uterine tamponade devices was limited and not generalizable. Rigorous economic evaluations based on updated effect estimates are needed.

## 1 INTRODUCTION

Obstetric hemorrhage is the leading cause of maternal mortality, contributing to 27.1% (uncertainty interval 19.9%–36.2%) of maternal deaths worldwide.^1^ The majority of these are postpartum hemorrhage (PPH), generally defined as blood loss of 500 mL or more within 24 hours after birth—a condition affecting an estimated 5% of all women who give birth.^2^, ^3^ Most maternal deaths due to PPH could be avoided by routine use of an effective uterotonic for PPH prophylaxis, as well as prompt and effective PPH management.^4^ Interventions recommended by WHO to manage PPH include fluid replacement, treatment with uterotonics and tranexamic acid, and use of non‐surgical (bimanual compression, uterine balloon tamponade [UBT], non‐pneumatic anti‐shock garment, external aortic compression) and surgical (compressive sutures, arterial ligation, or hysterectomy) interventions.^5^, ^6^ If bleeding persists despite treatment with uterotonic drugs, tranexamic acid, and non‐surgical interventions, surgical intervention should be used without delay.^4^

WHO’s 2012 guidelines on PPH prevention and management recommended that if a woman with PPH due to uterine atony does not respond to treatment using uterotonics (i.e., refractory PPH), or if uterotonics are unavailable, then UBT should be used (weak recommendation, very‐low‐quality evidence).^5^ In this situation, trained, skilled health personnel insert a balloon catheter inside the uterus that (when filled) applies hydrostatic pressure to reduce blood flow and facilitate clotting. In 2019, the WHO recommendation on UBT was prioritized for updating, in light of new evidence regarding the balance of risks and benefits of this intervention.^7^, ^8^

When guideline panels consider whether to recommend for or against the use of an intervention, consideration is given to a number of factors, including efficacy and safety, how feasible and acceptable the option is, whether it is cost‐effective, and the resources required to provide it.^9^ Cost‐effectiveness may vary depending on the setting, device type and cost. Even where the cost of using UBT is high, its use may be reasonable if it can lead to equally large health gains. Costs may also be offset by savings associated with a reduction in adverse outcomes.

To our knowledge, and at the date of submitting this manuscript, no previous systematic review has been conducted to identify and assess all available evidence related to the costs and cost‐effectiveness of uterine tamponade. This review aimed to determine the incremental resource inputs and cost‐effectiveness of using uterine tamponade as part of standard PPH care (versus comparators or usual measures) for the treatment of atonic PPH. By standard care, we mean the interventions recommended by WHO for PPH treatment.^5^, ^6^ However, we recognize that some studies may predate the WHO recommendation for a given intervention. The review was performed in the context of preparing the evidence base to update WHO's recommendation on UBT,^7^ to summarize the available evidence related to cost‐effectiveness of this intervention when used for the treatment of women with refractory PPH.

## 2 MATERIALS AND METHODS

### 2.1 Literature search

This systematic review was conducted according to a pre‐specified protocol, in line with the Preferred Reporting Items for Systematic Reviews and Meta‐Analyses (PRISMA) guidelines (see PRISMA checklist in File S1).^10^ As a review of publicly available literature, ethical approval was not required. We developed a search strategy (combining concepts and synonyms for the third stage of labor, uterine tamponade, PPH, and cost‐effectiveness) and on January 15, 2020 searched PubMed (January 1, 1980 to date of search), EMBASE (January 1, 1980) and the National Health Services Economic Evaluation (NHS EED) database (inception in 1995 to April 2, 2015, database closure) (search strategy shown in File S2). We also screened the reference lists of any included studies for systematic reviews related to UBT effectiveness.^11^ Eligible studies were economic evaluations (including full or partial economic evaluations, cost‐benefit analyses, cost‐effectiveness analyses, cost‐utility analyses, cost analyses, cost description studies) or effectiveness studies (such as trials) that provide cost or economic data. Studies were included if they related to the use of uterine tamponade compared with standard care or other uterine tamponade devices for the treatment of women with PPH in the third stage of labor (after vaginal birth or cesarean section), in any healthcare setting. The incremental cost‐effectiveness ratio was the primary outcome of interest, though we extracted all available data related to cost or cost‐effectiveness. Cost data were reported as described in the paper; no standardization or cost adjustment was used.

### 2.2 Data extraction

We adopted the Cochrane guidance for economic evaluations.^12^ Two reviewers (JV and AW) independently assessed the eligibility of recovered citations using the Covidence platform, with disagreements resolved through discussion or consultation with a third reviewer.^13^ A data extraction form was adapted from a 2019 systematic review of cost‐effectiveness of uterotonics by Lawrie et al.,^14^ which was adapted from NHS EED guidance.^15^ For each eligible study, two reviewers independently extracted data relating to study design (aim, design, setting, year, sources of costs and effectiveness data, analytical perspective, time horizon) and relevant outcomes (costs of treatment options considered, main findings). Quality of cost‐effectiveness studies was assessed with the Consensus Health Economic Criteria (CHEC) checklist, with disagreements resolved through discussion or consultation with other reviewers (see File S3).^16^ All extracted data and quality assessments were reviewed by a health economist (NS).

### 2.3 Data synthesis

**Figure 1:**
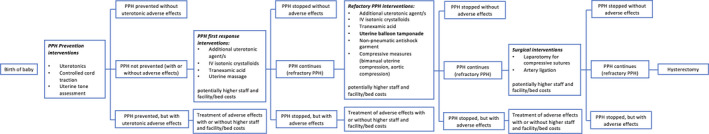
Diagram of possible cost consequences associated with using uterine tamponade in the management of atonic postpartum hemorrhage.

A conceptual framework was developed to clearly identify the role and possible cost consequences of using uterine tamponade in the management of atonic PPH (Fig. 1), informed by current WHO guidance on PPH prevention and management.^4^ Extracted data were summarized using tables, and brief narrative summaries of principal results and differences between studies were constructed. The currency and price year applicable to measures of costs in each study are reported alongside measures of costs, incremental costs and incremental cost‐effectiveness. We originally planned to consider subgroups by mode of birth, high versus low‐ and middle‐income countries and different uterine tamponade devices; however, these were not performed because of limited data.

## 3 RESULTS

### 3.1 Characteristics of included studies

**Figure 2:**
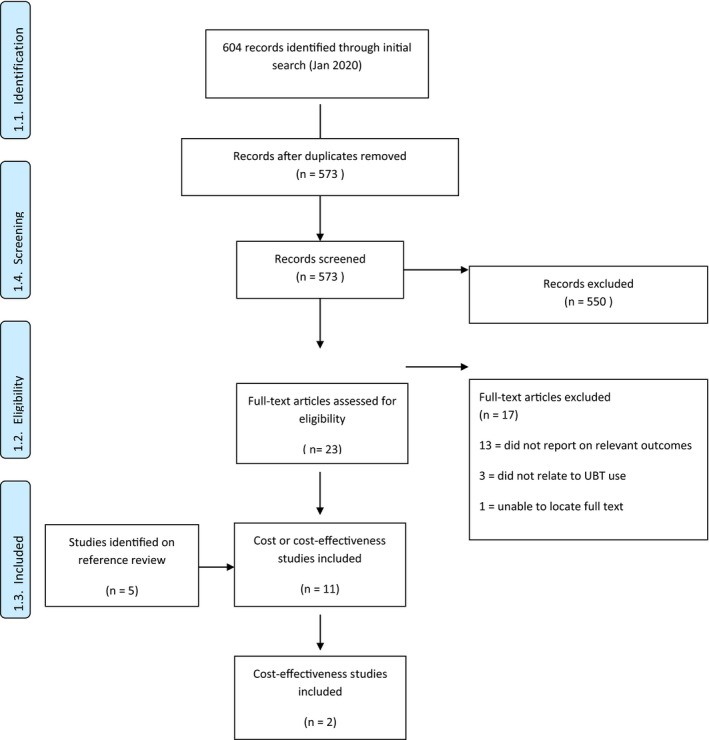
PRISMA flowchart.

In total, 573 unique records were identified and screened, of which 550 were excluded at title and abstract screening (Fig. 2). Of the 23 full texts reviewed, 13 did not report on relevant economic outcomes and three did not relate to UBT use. A healthcare technology brief on UBT was potentially eligible^17^; however, we were unable to obtain the full text of this report. Six studies were identified as eligible; on review of references a further five eligible studies were identified (11 studies in total) (Table 1).^18^, ^19^, ^20^, ^21^, ^22^, ^23^, ^24^, ^25^, ^26^, ^27^, ^28^ All studies provided some type of cost information on various tamponade devices. Four studies were case series,^18^, ^21^, ^26^, ^28^ three were randomized trials,^19^, ^24^, ^25^ one was a non‐randomized interventional study,^23^ one was a modelling study,^20^ and two were cost‐effectiveness analyses.^22^, ^27^ Studies were conducted in Benin, Egypt, India, Indonesia, Kenya (two studies), Mali, Nepal, Sierra Leone, Senegal, South Africa (two studies), and Turkey (the modelling study considered all of sub‐Saharan Africa, and the economic assessment considered all countries). Uterine tamponade devices described in these studies included UBT improvised devices (condom catheter); UBT purpose‐designed devices (Every Second Matters for Mothers and Babies (ESM)–UBT kit; Bakri balloon; Ellavi; Sinapi Biomedical, Stellenbosch, South Africa); uterine suction improvised devices (FG36 Levin stomach tube); and uterine suction purpose‐designed devices (published as Inpress, subsequently described as the Jada System by Alydia Health (Menlo Park, CA, USA).^29^

**Table 1:** Characteristics of included studies.

Included study	Study design	Country/ies	Relevance to this review	UBT device/s	Device cost
Burke 2016^18^	Prospective case series	Kenya, Sierra Leone, Senegal, and Nepal	Provides cost of a device	Condom uterine balloon tamponade kit (Every Second Matters for Mothers and Babies™–UBT)	“less than US$5”
Darwish 2017^24^	Randomized controlled trial	Egypt	Provides cost of two devices	Bakri balloon versus condom‐loaded Foley catheter	“The cost of BB at our country equals 2700 EP [US$172] while the cost of a single set of CLFC is just 10 EP [US$0.64].”^a^
Dumont 2017^19^	Randomized controlled trial	Benin and Mali	Provides cost of a device	Condom catheter	“Tablets of 200 μg misoprostol and UBT kits (including Foley catheter size 24, condom, 1‐litre bag of solute, needleless suture, 50 mL syringe, compresses, sterile gloves) were implemented in the participating centers (each kit costing US$10 but free of charge for the patients)”.
Herrick 2017^20^	Modeling study (morbidity and mortality impact of UBT)	Sub‐Saharan Africa	Provides cost of a device	Purpose‐designed devices	“UBT is still widely underutilised and unavailable in low‐ and middle‐income countries, largely because commercial devices are prohibitively expensive, ranging from $US125 to $350 for one‐time use.”
Hofmeyr 2019^25^	Randomized controlled trial (pilot)	South Africa	Provides cost of a device	FG36 Levin stomach tube (as a uterine suction device)	“…widely available at minimal cost (less than US$2 for FG36; less than US$0.30 for FG24).”
Kaya 2016^26^	Retrospective case series	Turkey	Provides cost of a device	Bakri balloon	“The cost of the Bakri balloon changes between 250 and 300 dollars in various countries”
Mishra 2019^21^	Prospective case series	India	Provides cost of a device	Chhattisgarh condom balloon device versus conventional condom uterine balloon device	“The average cost of C‐UBT use was Rs.400 (approximately $5), and that of CGB was Rs.120 (approximately $2).”
Mvundura 2017^b^	Cost‐effectiveness analysis	Kenya	Provides cost of a device Provides cost‐effectiveness estimate	Condom uterine balloon tamponade kit (Every Second Matters for Mothers and Babies™–UBT)	“The ESM‐UBT device was not commercially available at the time of the present study; hence, an assumed price of $5 was used in the analysis. This price was based on the cost of materials needed to assemble the device (a condom, string, catheter, and syringe). Modeling at a price assumption of $15 was also undertaken.”
Purwusonu 2016^23^	Non‐randomized prospective experimental study	Indonesia	Provides cost of a device	Vacuum‐induced tamponade device (Inpress)	“The vacuum‐induced tamponade device, manufactured by InPress Technologies, Inc, is low cost (less than $400), one piece, comes in a sterile package designed for one‐time use, and is made of medical‐grade silicone.”
Seligman 2006^c^	Economic assessment of PPH prevention and treatment interventions (including UBT)	Developing countries	Provides cost of a device Provides cost‐effectiveness estimate	“Balloon tamponade: condom and catheter preconnected, sterilized, and packed”	“No market price was available for the balloon tamponade. The estimated price ($6.00) is the sum of the price of a condom, catheter, 500 ml saline, and other materials. The price also accounts for the cost of prepackaging and sterilization.”
Theron 2018^28^	Prospective case series	South Africa	Provides cost of a device	“The free‐flow pressure controlled uterine balloon (Ellavi UBT; Sinapi biomedical)”	“The device is affordable for use in lesser resourced countries with a sales price from the factory of approximately US$6.”

^a^ Egyptian pounds converted to US$ at rate of 15.719 EGP to US$1 (UN Operational Rate of Exchange, April 2020).

^b^ Consensus Health Economic Criteria (CHEC) checklist assessment was high quality (17/19).

^c^ Consensus Health Economic Criteria (CHEC) checklist assessment was moderate quality (12/19).

Studies published between 2006 and 2019 quoted condom catheter devices or kits at US$0.64 to US$6, though in the 2017 trial by Dumont et al. a kit composed of 200‐μg misoprostol tablets, a Foley catheter, condom, 1‐L bag of solute, needleless suture, 50‐mL syringe, compresses and sterile gloves was quoted as costing US$10.^19^, ^21^, ^24^, ^27^ A 2016 paper quoted the pre‐designed ESM‐UBT was “less than US$5”, and Ellavi was quoted as approximately US$6 in a 2018 paper.^18^, ^22^, ^28^ A 2019 pilot trial evaluated the feasibility of using the FG36 Levin stomach tube as an improvised uterine suction device, which cost less than US$2.^25^ Purpose‐designed devices were significantly more expensive—Bakri balloon was quoted at US$250 to US$300 in a 2016 paper,^26^ Inpress (Alydia) device at less than US$400 in a 2016 paper,^23^ and “commercial devices” (not otherwise specified) ranging between US$125 and US$350 in a 2017 paper.^20^

The two cost‐effectiveness studies^22^, ^27^ used a model‐based approach to estimate the incremental costs of introducing UBT using a condom catheter device to treat PPH (see File S3). One was a cost‐effectiveness analysis on the introduction of a low‐cost UBT model (ESM‐UBT) to routine PPH management at health center and hospital levels for women giving birth in Kenya in 2015 (rated as high quality on CHEC).^22^ Cost data were obtained through interviews with staff at 30 purposely selected facilities in Kenya, and included medications, supplies, laboratory tests, time spent managing women with PPH, and training costs. The analysis took a health system perspective, and estimated costs for all women undergoing PPH in Kenya in a 1‐year period. The intervention (ESM‐UBT) was not commercially available; however, price assumptions of US$5 and US$15 were used. Estimates of the effects of UBT were derived from a 2016 multicenter case series study conducted in Kenya, Sierra Leone, Senegal, and Nepal (sample size 201 women).^18^ This study implemented a standardized ESM‐UBT package in 307 facilities across these four countries over a 29‐month period, and reported all‐cause maternal survival at 95%. The study used a decision tree model, considered a 1‐year time horizon for costs, and a lifetime horizon for benefits for women receiving the intervention (e.g. disability‐adjusted life‐years [DALYs] from deaths averted), did not include cost or benefit discounting, and performed multivariate probabilistic sensitivity analyses to test the impact of varying cost, coverage and outcome parameters. The analysis considered (1) the base case (current practice, where UBT was not used), (2) the availability of uterine packing at health centers for women with PPH before transfer to hospital, and (3) the same conditions as (1) and (2) plus the availability of ESM‐UBT at health centers or hospitals after uterotonic drugs and mechanical interventions had failed to stop PPH. It was assumed that only women who continued to experience PPH were transferred to hospital. The third scenario totaled an additional US$64,341 per annum across Kenya compared with the base case. With a US$5 price, the analysis found US$26 incremental cost per DALY averted (and less than US$41 per DALY averted in all sensitivity analyses), and with a US$15 price the analysis found US$40 incremental cost per DALY averted. This was described as highly cost‐effective, considering that Kenya’s GDP per capita was US$1358 in 2014 and the estimated opportunity cost of healthcare in Kenya was US$500–700 in 2015.^30^

The second study was a 2006 economic assessment of a number of PPH prevention and treatment interventions in developing countries only, including UBT (rated as moderate quality on CHEC).^27^ This study took a health system perspective; however, it included costs borne by patients and also took a societal perspective for the cost‐benefit analysis (DALYs averted were converted to dollars based on GDP per capita). It used an Excel‐based model (the details of which were not available) to estimate the cost‐effectiveness of these interventions, and univariate deterministic sensitivity analyses were performed for different protection rates, coverage rates, and prices of drugs and products, with point estimates and ranges for parameters derived from international sources and consultation with country experts in four countries. The “protection rate” of UBT against death was assumed to be 75%, referencing two case series studies conducted in Bangladesh and the UK.^31^, ^32^ In the study in Bangladesh, a condom catheter was used in 23 women with hemorrhagic shock due to PPH following atonic uterus or placenta accreta, all of whom ceased bleeding within 15 minutes.^31^ In the UK study, 27 women with uncontrolled PPH were managed by UBT using a Sengstaken‐Blakemore esophageal catheter, and bleeding ceased in 22 (81%) of the women.^32^ An estimated price of US$6 was used (condom, catheter, 500 mL saline, and other materials, including pre‐packaging and sterilization). Costs were estimated for the years 2006, 2010, and 2015; equating to an intervention (and cost) time horizon of 1, 5, and 10 years, with lifetime benefits included for women receiving the intervention in those time periods. Costs and benefits were not discounted. The authors reported that UBT was highly cost‐effective, associated with a cost of US$1.00 per DALY averted (the lowest among all considered interventions, and a maximum of US$1.06 in the sensitivity analysis), with a cost‐benefit ratio of US$1644.21.

## 4 DISCUSSION

Limited evidence is available regarding the cost‐effectiveness of UBT for the treatment of PPH, and no cost‐effectiveness evidence was found for other tamponade devices, such as suction tamponade. Some tamponade options cost between US$0.64 to US$6, including two purpose‐designed devices (ESM‐UBT and Ellavi), though other purpose‐designed devices cost more (US$125 to nearly US$400).

The two cost‐effectiveness analyses indicated that UBT using condom catheter is highly cost‐effective from a health system perspective (on the basis of a relatively low cost per DALY averted), but both studies used effect estimates derived from case series with relatively small sample sizes. Neither study used discounting of costs of benefits; however, given the acute and simultaneous nature of the intervention delivery, its cost and its impact (in particular the single year time‐horizon for costs in Mvundura et al.^22^) this is appropriate. For Seligman and Xingzhu,^27^ this is unlikely to influence the estimates of cost‐effectiveness, but may have meant that the total costs were overstated over their longer time projections. The cost‐effectiveness of UBT for the treatment of PPH is likely to vary across settings, depending on both setting‐specific costs and setting‐specific effectiveness (which may vary because of a range of factors including healthcare worker training and availability of auxiliary infrastructure and equipment). The two cost‐effectiveness analyses tested the sensitivity of different input costs, which provides some insight into how cost‐effectiveness might change between settings. However, these studies either did not undertake sensitivity analysis of effect estimates^22^ or tested variations of effectiveness between 50% and 75%,^27^ which may be optimistic. With point estimate effect sizes based on case series with relatively small sample sizes, this uncertainty in cost‐effectiveness estimates remains to be tested. Other key differences between the two studies related to scope and health outcomes—Mvundura et al.^22^ considered cost‐effectiveness in a single country over a 1‐year period and considered important health outcomes such as hospital transfers, hysterectomies, and death, as well as DALYs averted.^22^ Comparatively, Seligman and Xingzhu^27^ was an international assessment of less‐developed countries considering 1‐, 5‐ and 10‐year scenarios and focused primarily on PPH‐related deaths and DALYs averted.^27^

We identified no cost‐effectiveness analyses of purpose‐designed devices, which are generally more expensive and widely used in high‐resource settings. There is therefore insufficient evidence to conclude whether uterine tamponade is cost‐effective. To our knowledge, this is the first systematic review of cost‐effectiveness of uterine tamponade for the treatment of PPH. Strengths of this review include a standard protocol and an extensive systematic search across multiple databases. Despite our efforts, limited data are available on this question—although the citation by Hayes Inc.^17^ is promising, a limitation of this review is that we were unable to obtain this report despite contacting the authors. We were unable to perform pre‐specified subgroup analyses (mode of birth, income level of countries, different tamponade devices) because of limited data. Our review is a systematic review of available economic analyses, but is itself not a cost‐effectiveness analysis. When further evidence becomes available, it is therefore likely that the conclusions of this review will change.

There are inherent limitations in basing cost‐effectiveness assessments on effect‐estimates‐derived observational studies (such as case series). Although observational studies may suggest substantial benefit, good‐quality randomized trials are required to establish reliable estimates of benefits and harms. In the case of UBT, WHO’s 2012 weak recommendation in favor of UBT for refractory PPH was supported only by observational evidence (no trials were available at that time).^5^ However, to our knowledge only two trials have compared UBT with no UBT after vaginal birth (116 women and 240 women, respectively), suggesting that the benefits and harms of this intervention are not yet known.^19^, ^33^ Furthermore, a 2019 stepped‐wedge, cluster‐randomized trial assessed the effectiveness of introducing condom catheter UBT as an option for the treatment of refractory PPH after vaginal birth in 18 hospitals in Uganda, Egypt, and Senegal.^34^ The trial authors reported that UBT introduction was associated with a significant increase in the composite outcome of PPH‐related invasive procedures and/or maternal death. It is perhaps unsurprising that cost‐effectiveness analyses based on optimistic estimates of benefits and harms would produce favorable results. Further research is evidently required, particularly the need for robust cost‐effective analyses that are based on effect estimates derived from randomized trials of uterine tamponade interventions, for both improvised and purpose‐designed devices. These analyses will need to consider the considerable differences in contexts and costs associated with introducing and/or scaling up uterine tamponade programs. Such findings would provide critical additional information to guide clinicians, policymakers, and other stakeholders.

## 5 CONCLUSION

There is insufficient evidence to reliably determine the cost‐effectiveness of uterine tamponade for the treatment of PPH. It is, however, likely that the cost‐effectiveness of this intervention would differ in different settings and with different tamponade devices. In light of the widespread use of this intervention for refractory PPH, more rigorous economic evaluations based on reliable effect estimates are needed.

## AUTHOR CONTRIBUTIONS

The concept for this article was conceived by JPV and OTO. The protocol was drafted by JPV, with input from ANW, NS, and OTO. Literature screening, data extraction, and quality assessment were performed by JPV, ANW, and NS. All authors contributed to the analysis, interpretation, and write up. This article represents the views of the named authors only, and not the views of their institutions.

## CONFLICTS OF INTEREST

The authors have no conflicts of interest.

## Supporting information


**File S1**. PRISMA checklist.

**File S2**. Search strategy.

**File S3**. Extracted data from cost‐effectiveness studies and CHEC quality assessment.
