# Supplementary material for: Cost‐effectiveness of uterine tamponade devices for the treatment of postpartum hemorrhage: A systematic review
Source: Int J Gynaecol Obstet. 2020 Oct 30;151(3):333–40. doi: 10.1002/ijgo.13393 (PMC7756424; doi:10.1002/ijgo.13393)
Supplement: Supplementary file 2 — File S2. Search strategy. [file IJGO-151-333-s002.docx]

Cost-effectiveness of uterine balloon tamponade for the treatment of postpartum haemorrhage

Search strategy

15 Jan 2020

**PubMed**

| **Concept** | **Search string** | **Results**  **15 Jan 2020** |
| --- | --- | --- |
| 1-Third stage of labour | “Postpartum Period”[Mesh] OR "Postnatal Care"[Mesh] OR "Delivery, Obstetric"[Mesh] OR "Parturition"[Mesh] OR "Labor, Obstetric"[Mesh] OR “Third stage”[tiab] OR “third stage of labour”[tiab] OR “third stage of labor”[tiab] OR “3^rd^ stage”[tiab] OR “3^rd^ stage of labor”[tiab] OR “Postpartum”[tiab] OR “post-partum”[tiab] OR “post partum”[tiab] OR “postnatal”[tiab] OR “post-natal”[tiab] OR “post natal”[tiab] OR “labour”[tiab] OR “labor”[tiab] OR “laboring”[tiab] OR “laboring”[tiab] OR “birth”[tiab] OR “births” [tiab] OR “childbirth”[tiab] OR “childbirths”[tiab] OR “child birth”[tiab] OR “child births”[tiab] OR “delivery”[tiab] OR “deliveries”[tiab] OR “obstetric delivery”[tiab] OR "obstetric deliveries”[tiab] | 982718 |
| 2-Uterine tamponade | “uterine balloon tamponade”[mesh] OR "uterus"[mesh] OR "Uterus"[tiab] OR "uterine"[tiab] OR "intrauterine"[tiab] OR "intra uterine"[tiab] OR “intra-uterine”[tiab] OR “uterine balloon tamponade”[tiab] OR “UBT”[tiab] OR “inpress”[tiab] OR “Bakri”[tiab] OR “Belfort Dildy”[tiab] OR “BT Cath”[tiab] OR “ebb balloon”[tiab] OR “ebb balloons”[tiab] OR “ebb tamponade”[tiab] OR “el menia”[tiab] OR “Rusch”[tiab] OR “Sengstaken Blakemore”[tiab] OR “Sengstaken”[tiab] OR “Blakemore”[tiab] OR “Sengstaken-Blakemore”[tiab] OR "catheters"[Mesh] OR “foley”[tiab] OR “foley's”[tiab] OR “foleys”[tiab] OR “foley catheter”[tiab] OR “foley’s catheter”[tiab] OR “foleys catheter”[tiab] OR “foley catheters”[tiab] OR “foley’s catheters”[tiab] OR “foleys catheters”[tiab] OR “balloon”[tiab] OR “balloons”[tiab] OR “tamponade”[tiab] OR “tamponades”[tiab] OR “condom”[tiab] OR “condoms”[tiab] OR “condoms”[mesh] OR “balloon occlusion“[mesh] OR “balloon occlusion”[tiab] OR “dilatation”[tiab] OR “dilation”[tiab] OR "fluid filled" [tiab] OR “gauze”[tiab] OR “hydrostatic”[tiab] OR “packing”[tiab] OR “sponge”[tiab] OR “sponges”[tiab] OR “Dilatation”[mesh] OR "Surgical Sponges"[mesh] OR "Gloves, Surgical"[Mesh] OR "Pressure"[Mesh] OR “glove*”[tiab] OR “vacuum induced”[tiab] OR “compression”[tiab] OR “haemostatic techniques”[tiab] OR “hemostatic techniques”[tiab] | 737773 |
| 3-haemorrhage | "Postpartum Hemorrhage"[Mesh] OR "Shock, Hemorrhagic"[Mesh] OR "Uterine Inertia" [mesh] OR "Uterine Hemorrhage" [mesh] OR "Abruptio Placentae"[mesh] OR "Blood Loss, Surgical"[mesh] OR "Blood Transfusion"[mesh] OR "Placenta Accreta" [mesh] OR "Placenta Previa" [mesh] OR “uterine inversion”[mesh] OR "Uterine Artery Embolization"[Mesh] OR "Uterine Contraction"[Mesh] OR "Obstetric Labor Complications"[Mesh] OR "Postpartum Hemorrhage" [tiab] OR “PPH”[tiab] OR “Abruptio Placentae”[tiab] OR “abruption”[tiab] OR "surgical blood loss"[tiab] OR “blood loss”[tiab] OR "blood transfusion"[tiab] OR “placenta accreta”[tiab] OR “placenta previa”[tiab] OR “shock”[tiab] OR “haemorrhagic shock”[tiab] OR “hemorrhagic shock”[tiab] OR “placental accreta”[tiab] OR “placenta previa”[tiab] OR “placenta praevia”[tiab] OR “placental previa”[tiab] OR “placental praevia”[tiab] OR “postpartum hemorrhage”[tiab] OR “postpartum haemorrhage”[tiab] OR “post partum hemorrhage”[tiab] OR “post partum haemorrhage”[tiab] OR “post-partum hemorrhage”[tiab] OR “post-partum haemorrhage”[tiab] OR “atony”[tiab] OR “atonic”[tiab] OR "atonic uterus"[tiab] OR "uterine atony"[tiab] OR "uterine inertia"[tiab] OR “uterine bleeding”[tiab] OR “uterine hemorrhage”[tiab] OR “uterine haemorrhage”[tiab] OR “uterus inversion”[tiab] OR “uterine artery embolization”[tiab] OR “uterine contraction”[tiab] OR “labor complication”[tiab] OR “labour complication”[tiab] OR “delivery complication”[tiab] | 421891 |
| 4 – cost effectiveness studies | "Cost-Benefit Analysis"[Mesh] OR "Costs and Cost Analysis"[Mesh] OR "Cost of Illness"[Mesh] OR "Cost Savings"[Mesh] OR "Health Care Costs"[Mesh] OR "Hospital Costs"[Mesh] OR "Health Expenditures"[Mesh] OR “cost-benefit”[tiab] OR “cost analysis”[tiab] OR “cost of illness”[tiab] OR “cost saving”[tiab] OR “health cost”[tiab] OR “health costs”[tiab] OR “health expenditure”[tiab] OR “economic evaluation” [tiab] OR “cost-effectiveness”[tiab] OR “cost effectiveness”[tiab] OR “cost-effective”[tiab] OR “cost effective”[tiab] OR “cost-utility”[tiab] OR “cost utility”[tiab] OR “cost description”[tiab] OR “economic analysis”[tiab] OR “budget impact”[tiab] OR “cost”[tiab] OR “economic”[tiab] OR “budget”[tiab] OR “economic evaluation”[tiab] OR “full economic evaluation”[tiab] OR “partial economic evaluation”[tiab] | 711094 |
|  | 1+2+3+4 | 438 |
|  | Publication date 1/1/1980 onwards | 430 |

EMBASE

| **Concept** | **Search string** | **Results**  **15 Jan 2020** |
| --- | --- | --- |
| 1-Third stage of labour | 'Postpartum'/exp OR ‘puerperium’/exp OR 'Postnatal Care'/exp OR ' obstetric delivery'/exp OR 'birth'/exp OR 'obstetric procedure'/exp OR ‘labor’/exp OR ‘labor complication’/exp OR ‘childbirth’/exp OR ‘labor stage 3’/exp OR ‘pregnancy’/exp OR ‘Third stage’:ti,ab OR ‘third stage of labour’:ti,ab OR ‘third stage of labor’:ti,ab OR ‘3rd stage’:ti,ab OR ‘3rd stage of labor’:ti,ab OR ‘Postpartum’:ti,ab OR ‘post-partum’:ti,ab OR ‘post partum’:ti,ab OR ‘postnatal’:ti,ab OR ‘post-natal’:ti,ab OR ‘post natal’:ti,ab OR ‘labour’:ti,ab OR ‘labor’:ti,ab OR ‘laboring’:ti,ab OR ‘laboring’:ti,ab OR ‘birth’:ti,ab OR ‘births’:ti,ab OR ‘childbirth’:ti,ab OR ‘childbirths’:ti,ab OR ‘child birth’:ti,ab OR ‘child births’:ti,ab OR ‘delivery’:ti,ab OR ‘deliveries’:ti,ab OR ‘obstetric delivery’:ti,ab OR ‘obstetric deliveries’:ti,ab | 2057493 |
| 2-Uterine tamponade | 'uterus'/exp OR ‘Uterus’:ti,ab OR ‘uterine’:ti,ab OR ‘intrauterine’:ti,ab OR ‘intra uterine’:ti,ab OR ‘intra-uterine’:ti,ab OR 'catheters'/exp OR ‘catheterization’/exp OR ‘foley balloon catheter’/exp OR ‘balloon catheter’/exp OR ‘balloon dilatation’/exp OR ‘balloon pump’/exp OR ‘intrauterine balloon’/exp OR ‘Sengstaken Blakemore tube’/exp OR ‘condom catheter’/exp OR ‘condom’/exp OR ‘balloon occlusion’/exp OR ‘occlusion balloon catheter’/exp OR ‘dilatation’/exp OR ‘balloon dilatation’/exp OR ‘gauze’/exp OR ‘gauze dressing’/exp OR ‘hydrostatic pressure’/exp OR ‘surgical sponge’/exp OR ‘surgical glove’/exp OR 'pressure'/exp OR ‘uterine balloon tamponade’:ti,ab OR ‘UBT’:ti,ab OR ‘inpress’:ti,ab OR ‘Bakri’:ti,ab OR ‘Belfort Dildy’:ti,ab OR ‘BT Cath’:ti,ab OR ‘ebb balloon’:ti,ab OR ‘ebb balloons’:ti,ab OR ‘ebb tamponade’:ti,ab OR ‘el menia’:ti,ab OR ‘Rusch’:ti,ab OR ‘Sengstaken Blakemore’:ti,ab OR ‘Sengstaken’:ti,ab OR ‘Blakemore’:ti,ab OR ‘Sengstaken-Blakemore’:ti,ab OR ‘foley’:ti,ab OR ‘foleys’:ti,ab OR ‘foley catheter’:ti,ab OR ‘foleys catheter’:ti,ab OR ‘foley catheters’:ti,ab OR ‘foleys catheters’:ti,ab OR ‘balloon’:ti,ab OR ‘balloons’:ti,ab OR ‘tamponade’:ti,ab OR ‘tamponades’:ti,ab OR ‘condom’:ti,ab OR ‘condoms’:ti,ab 'balloon occlusion':ti,ab OR ‘dilatation’:ti,ab OR ‘dilation’:ti,ab OR ‘fluid filled’:ti,ab OR ‘gauze’:ti,ab OR ‘hydrostatic’:ti,ab OR ‘packing’:ti,ab OR ‘sponge’:ti,ab OR ‘sponges’:ti,ab OR ‘glove*’:ti,ab OR "vacuum induced":ti,ab OR ‘compression’:ti,ab OR "hemostatic techniques":ti,ab | 359394 |
| 3-haemorrhage | 'Postpartum Hemorrhage'/exp OR “Hemorrhagic shock'/exp OR 'uterus bleeding'/exp OR 'solutio placentae'/exp OR ‘bleeding’/exp OR 'operative blood loss'/exp OR 'Blood Transfusion'/exp OR 'Placenta Accreta '/exp OR 'Placenta Previa '/exp OR 'uterus inversion'/exp OR 'uterine artery embolization'/exp OR 'uterus contraction'/exp OR ‘abruptio placentae’:ti,ab OR ‘abruption’:ti,ab OR ‘surgical blood loss’:ti,ab OR ‘blood loss’:ti,ab OR ‘blood transfusion’:ti,ab OR ‘placenta accreta’:ti,ab OR ‘placenta previa’:ti,ab OR ‘shock’:ti,ab OR ‘haemorrhagic shock’:ti,ab OR ‘hemorrhagic shock’:ti,ab OR ‘placental accreta’:ti,ab OR ‘placenta previa’:ti,ab OR ‘placenta praevia’:ti,ab OR ‘placental previa’:ti,ab OR ‘placental praevia’:ti,ab OR ‘PPH’:ti,ab OR ‘postpartum hemorrhage’:ti,ab OR ‘postpartum haemorrhage’:ti,ab OR ‘post partum hemorrhage’:ti,ab OR ‘post partum haemorrhage’:ti,ab OR ‘post-partum hemorrhage’:ti,ab OR ‘post-partum haemorrhage’:ti,ab OR ‘atony’:ti,ab OR ‘atonic’:ti,ab OR ‘atonic uterus’:ti,ab OR ‘uterine atony’:ti,ab OR ‘uterine inertia’:ti,ab OR ‘uterine bleeding’:ti,ab OR ‘uterine hemorrhage’:ti,ab OR ‘uterine haemorrhage’:ti,ab OR ‘labor complication’:ti,ab OR ‘delivery complication’:ti,ab OR ‘labour complication’:ti,ab OR ‘uterus inversion’:ti,ab OR ‘uterine artery embolization’:ti,ab OR ‘uterine contraction’:ti,ab | 1308879 |
|  | 1 AND 2 AND 3 | 2348 |
|  |  |  |
| 4 – cost effectiveness studies | 'Cost Benefit Analysis'/exp OR 'Cost'/exp OR 'Cost of Illness'/exp OR 'Cost control'/exp OR 'Health Care Cost'/exp OR 'Hospital Cost'/exp OR ‘cost-benefit’:ti,ab OR ‘cost analysis’:ti,ab OR ‘cost of illness’:ti,ab OR ‘cost saving’:ti,ab OR ‘health cost’:ti,ab OR ‘health costs’:ti,ab OR ‘health expenditure’:ti,ab OR ‘economic evaluation’:ti,ab OR ‘cost-effectiveness’:ti,ab OR ‘cost effectiveness’:ti,ab OR ‘cost-effective’:ti,ab OR ‘cost effective’:ti,ab OR ‘cost-utility’:ti,ab OR ‘cost utility’:ti,ab OR ‘cost description’:ti,ab OR ‘economic analysis’:ti,ab OR ‘budget impact’:ti,ab OR ‘cost’:ti,ab OR ‘economic’:ti,ab OR ‘budget’:ti,ab OR ‘economic evaluation’:ti,ab OR ‘full economic evaluation’:ti,ab OR ‘partial economic evaluation’:ti,ab | 1027156 |
|  | 1 + 2 +3 + 4 | 158 |
|  | 1/1/1980 onwards | 158 |

NHS EED

| **Concept** | **Search string** | **Results**  **15 Jan 2020** |
| --- | --- | --- |
| 1-Third stage of labour | MeSH DESCRIPTOR, EXPLODE ALL TREES  postpartum period; postnatal care; parturition; delivery obstetric;  Labor Stage, Third; Labor, Obstetric; Obstetric Labor Complications  Any field:  (third stage) OR (third stage of labour) OR (third stage of labor) OR  (3rd stage) OR (3rd stage of labor) OR (Postpartum) OR (post-partum) OR (post partum) OR (postnatal) OR (post-natal) OR (post natal) OR (labour) OR (labor) OR (birth) OR (births) OR (childbirth) OR (childbirths) OR (child birth) OR (child births) OR (delivery) OR (deliveries) OR (obstetric delivery) OR (obstetric deliveries) | 3563 |
| 2-Uterine tamponade | MeSH DESCRIPTORS EXPLODE ALL TREES  Uterine Balloon Tamponade, Uterus, Uterine Inversion, Catheterization, Catheters, Urinary Catheterization, Urinary Catheters, Balloon Occlusion, Condoms, Dilatation, Hydrostatic Pressure, Gloves, Surgical, Gloves, Protective, Pressure  Any field:  (Uterine balloon tamponade) OR (UBT) OR (Inpress) OR (Bakri) OR (Belfort Dildy) OR (BT Cath) OR (Ebb balloon) OR (Tamponade) OR (El menia) OR (Rusch) OR (Sengstaken) OR (Sengstaken Blakemore) OR (Foley) OR (Foleys) OR (Foley catheter) OR (Foleys catheter) OR (Balloon) OR (Condom) OR (balloon occlusion) OR (dilatation) OR (dilation) OR (fluid filled) OR (gauze) OR (hydrostatic) OR (packing) OR (sponge*) OR (glove*) OR (vacuum induced) OR (compression) OR (hemostatic technique*) | 2229 |
| 3-haemorrhage | MeSH DESCRIPTOR EXPLODE ALL TREES  Postpartum Hemorrhage; Blood Loss, Surgical; Uterine Hemorrhage; Abruptio Placentae; Shock, Hemorrhagic; Postoperative Hemorrhage; Blood Transfusion; Placenta Accreta; Uterine Inversion; Uterine Artery Embolization; Uterine Inertia  Any field:  (abruptio placentae) OR (abruption) OR (surgical blood loss) OR (blood loss) OR (blood transfusion) OR (placenta accreta) OR (placenta previa) OR (shock) OR (haemorrhagic shock) OR (hemorrhagic shock) OR (placental accreta) OR (placenta previa) OR (placenta praevia) OR (placental previa) OR (placental praevia) OR (PPH) OR (postpartum hemorrhage) OR (postpartum haemorrhage) OR (post partum hemorrhage) OR (post partum haemorrhage) OR (post-partum hemorrhage) OR (post-partum haemorrhage) OR (atony) OR (atonic) OR (atonic uterus) OR (uterine atony) OR (uterine inertia) OR (uterine bleeding) OR (uterine hemorrhage) OR (uterine haemorrhage) OR (labor complication) OR (delivery complication) OR (labour complication) OR (uterus inversion) OR (uterine artery embolization) OR (uterine contraction) | 1384 |
|  | 1 AND 2 AND 3 | 16 |
|  | 1/1/1980 onwards | 16 |
